# Machine Learning Prediction Model for Carotid‐Femoral Pulse Wave Velocity in Cardiovascular Health Assessments

**DOI:** 10.1111/jch.70049

**Published:** 2025-05-26

**Authors:** Minglong Xin, Vipin Kumar, Megumi Narisawa, Chunzi Jin, Wenhu Xu, Xian Wu Cheng

**Affiliations:** ^1^ Department of Cardiology and Hypertension Jilin Provincial Key Laboratory of Stress and Cardiovascular Disease Yanbian University Hospital Yanji Jilin P. R. China; ^2^ Department of Cardiology Nagoya University Graduate School of Medicine Nagoya Aichi Japan

**Keywords:** cardiovascular health, carotid artery, machine learning, pulse wave velocity

Cardiovascular disease (CVD) remains a major global health concern and consistently ranks as the leading cause of mortality worldwide. Among the key pathophysiological factors that drive the progression of CVD, vascular health and structural changes in the arterial wall play crucial roles [1]. Aortic stiffness, in particular, is known as a significant and independent predictor of cardiovascular events and mortality, retaining its prognostic value even after adjustment for traditional risk factors. Aortic stiffness refers to the loss of the aortic wall's elasticity, which occurs naturally with age but is accelerated by conditions such as hypertension, diabetes, dyslipidemia, and chronic inflammation [2]. The pathophysiological consequences of increased aortic stiffness are complex; the stiffness increases systolic blood pressure (SBP) while decreasing diastolic blood pressure (DBP), leading to increased pulse pressure and left ventricular afterload (Figure 1). These hemodynamic changes promote left ventricular hypertrophy and significantly increase the risk of cardiovascular events [1]. Monitoring arterial stiffness can detect changes in vascular function earlier and predict the risk of CVD, potentially allowing preventive interventions before clinical manifestations occur.

**FIGURE 1:**
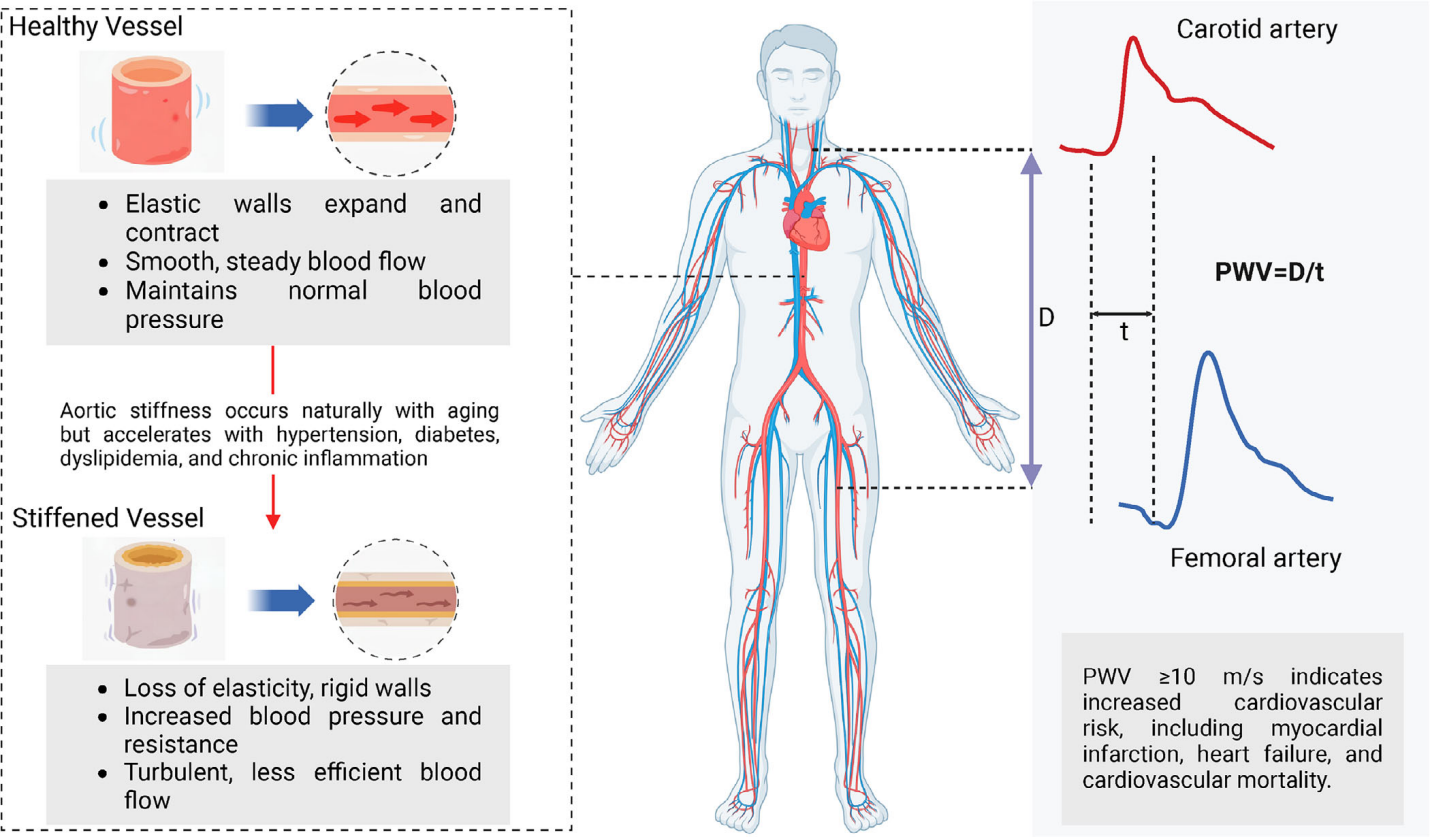
Measurement of carotid‐femoral pulse wave velocity in healthy and diseased arteries.

The development of CVD is a long‐term process, and early detection and intervention can prevent disease progression, reduce medical costs, and lower mortality rates. In this context, machine learning methods offer a promising approach to the detection of early signs of CVD and potentially improve cardiovascular health. For example, an algorithm for estimating the size of an abdominal aortic aneurysm that uses deep learning models to analyze pressure waves from the carotid, brachial, and femoral arteries was proposed in 2021 [3]. In vascular aging research, an artificial neural network was used to estimate carotid‐femoral pulse wave velocity (cf‐PWV), but that approach required central carotid pressure wave data and additional medical information such as chronological age [4]. The cf‐PWV, widely considered the gold standard for assessing atherosclerosis, plays a central role in estimations of the cf‐PWV [5]. Guidelines issued by the European Society of Cardiology and the European Society of Hypertension incorporate the cf‐PWV as a recommended parameter for cardiovascular risk assessments, with values >10 m/s indicating an increased risk [6]. Elevated cf‐PWV has been established as independently associated with increased risks of myocardial infarction, heart failure, and cardiovascular mortality over and above traditional cardiovascular risk factors [1, 5].

The study by Chen et al. in this issue of the *Journal of Clinical Hypertension* [7] presents a significant advance in cardiovascular risk assessment based on the development of machine learning models to predict the cf‐PWV. Chen et al. meticulously constructed and validated several machine learning models using data from the Northern Shanghai Study [8], a prospective, community‐based cohort of 2709 participants aged ≥65 years examined in 2013–2022 [7]. Recognizing that the traditional cf‐PWV measurements require specialized equipment and trained personnel (barriers to widespread clinical use), they sought to develop predictive models based on more accessible clinical parameters [7].

In their study, feature selection was guided by Pearson correlation coefficients, which identified the following as key predictors: the brachial‐ankle pulse wave velocity (ba‐PWV), age, sex, right‐brachial SBP, and right‐brachial DBP. The dataset was divided into 80% for training and 20% for testing. The study's methodological strength is evident in its systematic approach to model development. Five machine learning models were evaluated: linear regression, support vector regression, gradient boosting, random forest, and k‐nearest neighbor. Among them, the linear regression model demonstrated superior regression performance, achieving the lowest root mean square error at 1.383 m/s, the highest *R* ^2^ at 0.507, and the lowest percentage error at 15.049%. For the classification task of identifying individuals with cf‐PWV >10 m/s, which is a clinically significant threshold indicating increased cardiovascular risk, the gradient boosting model excelled with a 0.8449 area under the curve, 0.7856 accuracy, 0.7067 precision, and a recall value at 0.5856 [7]. This approach provides a practical and scalable solution to expand access to cf‐PWV‐based cardiovascular risk assessment, particularly in resource‐limited or community settings, thus directly addressing the barriers of specialized equipment and trained personnel requirements that have historically limited the widespread clinical implementation of cf‐PWV measurement.

In the Chen et al. study, a Cox proportional hazards model revealed that machine learning‐predicted cf‐PWV values were significantly associated with mortality risk, even when the ba‐PWV lost predictive power in a smaller validation dataset (20%). This finding supports the clinical utility of the predictive model, which also allows physicians to estimate cf‐PWV values without specialized equipment, facilitating broader cardiovascular risk screening. Highly predicted cf‐PWV values can help identify individuals who may benefit from more precise measurements and targeted interventions, thereby improving healthcare efficiency by providing specialized cf‐PWV testing for high‐risk patients. Importantly, the Cox proportional hazards analysis further confirmed the clinical validity of these machine learning‐based predictions, finding significant associations between predicted cf‐PWV values and mortality risk. The linear regression model yielded a χ^2^ value at 8.206 (*p* = 0.004), and the gradient boosting model yielded a χ^2^ value at 3.965 (*p* = 0.046), both approaching the association strength of actual cf‐PWV measurements (*χ* ^2^ =  17.882, *p* < 0.001) [7].

Feature selection guided by Pearson correlation coefficients in the Chen et al. study identified the following as the most important predictors of cf‐PWV: the ba‐PWV, age, sex, right‐brachial SBP, and right‐brachial DBP. These parameters are readily available in the primary care settings and provide a convenient set of clinical indicators. Despite the availability of more complex models, the results obtained by Chen and colleagues suggest that a well‐designed linear model with strategically selected features can be highly effective for this prediction task, offering a promising approach to expand access to cardiovascular risk assessments in resource‐limited settings.

Moreover, a key feature of the Chen study was the use of the Python package SHAP (SHapley Additive exPlanations) to analyze the contributions of individual features. Their SHAP analysis identified ba‐PWV as the dominant predictor of cf‐PWV, with a positive correlation indicating that higher ba‐PWV values lead to higher cf‐PWV predictions. Right‐brachial SBP was confirmed as a significant positive predictor, strengthening the physiological link between peripheral vascular measures and central arterial stiffness.

Although the Chen et al. study has several strengths, several considerations that deserve attention. The study population was limited to older participants (≥65 years) from northern Shanghai, which may limit the findings' generalizability to younger populations and/or those from different geographic or ethnic backgrounds. Future validation in more diverse cohorts would help strengthen the broader applicability of these models. In addition, although the models demonstrated strong performance metrics, an approx. A 15% prediction error remains that clinicians should be aware of when using these tools in practice. This margin of error is likely acceptable for initial risk screening and triage purposes, but it may necessitate follow‐up with direct cf‐PWV measurements in cases where a precise assessment is critical, such as borderline‐risk patients and those with complex comorbidities. Incorporating machine learning to predict cardiovascular risks into a clinical workflow also raises important questions. As healthcare systems increasingly adopt artificial intelligence‐based decision support, careful attention must be paid to how these predictions are presented to clinicians and patients, how they influence clinical decision‐making, and how they are integrated with traditional approaches to risk assessment.

The recommendation by Dr. Chen et al. to use predictive models to identify patients with high predicted cf‐PWV values for more accurate measurements represents a reasonable, stepwise approach that leverages the power of these models while maintaining the gold standard for definitive assessment. Looking forward, their study opens several promising directions for further exploration. Investigations of how changes in predicted cf‐PWV correlate with clinical outcomes over time could provide valuable insights into the progression of atherosclerosis [2, 9, 10], and exploring how these prediction models could be combined with other cardiovascular risk assessment tools could improve comprehensive risk stratification approaches.

In conclusion, Chen et al. have significantly contributed to cardiovascular risk assessments by demonstrating the feasibility and clinical relevance of machine learning‐based cf‐PWV prediction models. Their work effectively bridges the gap between dedicated arterial stiffness measurements and practical clinical applications, potentially broadening access to this important cardiovascular risk marker. This approach could be integrated with existing cardiovascular risk assessment tools in primary care settings, enhancing current risk stratification methodologies without requiring substantial additional resources or specialized training. As healthcare continues to adopt data‐driven approaches, the Chen et al. study is a compelling example of how machine learning can address practical clinical challenges while remaining closely aligned with established physiological principles and clinical outcomes.

## Author Contributions

Minglong Xin wrote the first draft of the manuscript. Vipin Kumar and Megumi Narisawa drafted the figure. Chunzi Jin and Wenhu Xu edited the manuscript. Xian Wu Cheng handled the funding and supervision.

## Conflicts of Interest

The authors declare no conflicts of interest.

## Data Availability

The authors have nothing to report.
